# Case Report: Multiple skeletal lesions as the initial presentation of primary hyperparathyroidism: A case of parathyroid adenoma mimicking malignancy

**DOI:** 10.3389/fendo.2026.1745014

**Published:** 2026-02-16

**Authors:** Nanxi Li, Xi Yang, Jiuyang Chen, Xing Wei, Wenxiu Yao

**Affiliations:** 1 Department of Thoracic Medical Oncology, Sichuan Clinical Research Center for Cancer, Sichuan Cancer Hospital & Institute, Sichuan Cancer Center, School of Medicine, University of Electronic Science and Technology of China, Chengdu, Sichuan, China; 2 Department of Thoracic Medical Oncology, Sichuan Clinical Research Center for Cancer, Sichuan Cancer Hospital & Graduate School, Chengdu Medical College, Chengdu, Sichuan, China; 3 Department of Thoracic Surgery, Sichuan Clinical Research Center for Cancer, Sichuan Cancer Hospital & Institute, Sichuan Cancer Center, University of Electronic Science and Technology of China, Chengdu, China

**Keywords:** endocrine disorders, osteolytic lesions, parathyroid adenoma, parathyroidectomy, primary hyperparathyroidism

## Abstract

**Background:**

Primary hyperparathyroidism (PHPT) is a relatively common endocrine disorder, although advanced skeletal manifestations mimicking metastatic malignancy are uncommon, characterized by autonomous oversecretion of parathyroid hormone.

**Case presentation:**

We report the case of a 67-year-old female who presented with cough and chest pain and was later found to have extensive osteolytic lesions initially suspected as malignant bone metastases. A multidisciplinary evaluation, including laboratory testing and advanced imaging, was performed. The evaluation revealed primary hyperparathyroidism due to a parathyroid adenoma. Surgical resection of the adenoma normalized serum calcium and parathyroid hormone levels, leading to marked symptomatic improvement. By the last follow-up, her pain had significantly improved, and parathyroid hormone levels had normalized.

**Conclusions:**

This case highlights the importance of considering endocrine etiologies when evaluating patients with atypical bone lesions, particularly in patients presenting with atypical respiratory symptoms and thoracic imaging findings that closely mimic primary lung malignancy with bone metastases.

## Introduction

Primary hyperparathyroidism (PHPT) is a relatively common endocrine disorder, characterized by autonomous oversecretion of parathyroid hormone (PTH) due to parathyroid adenoma, hyperplasia, or carcinoma. Although most patients present with asymptomatic or mild hypercalcemia, advanced skeletal manifestations mimicking metastatic malignancy are uncommon. Patients typically present with hypercalcemia resulting from elevated PTH levels (1, 2). The prevalence of PHPT ranges from 4 to 820 cases per million, with a male-to-female ratio of approximately 1:3. Although most patients are postmenopausal, the disease can manifest at any age. Hyperparathyroidism is most commonly caused by a solitary parathyroid adenoma, accounting for approximately 78%–92% of cases. Osteolytic lesions involving the cortical bone may develop and occasionally lead to pathological fractures (3). Given its rarity and nonspecific presentation, PHPT is frequently overlooked in clinical practice. Thus, with this report, we aimed to document the diagnosis and treatment of a 67-year-old female with PHPT and multiple osteolytic bone lesions.

## Case presentation

### Patient information

A 67-year-old woman presented with a persistent cough, sputum production, and left-sided rib pain, which worsened with weightlifting and compression and had persisted for over a month. After one week of symptomatic anti-inflammatory therapy, her symptoms showed no significant improvement. The patient had no notable medical antecedents, no history of smoking or alcohol consumption, no family history of hereditary conditions or calcium-related metabolic disorders, and no history of kidney stones or fractures. On admission, her vital signs were as follows: blood pressure, 105/65 mmHg; height, 158 cm; and weight, 47 kg.

### Clinical findings

**Figure 1:**
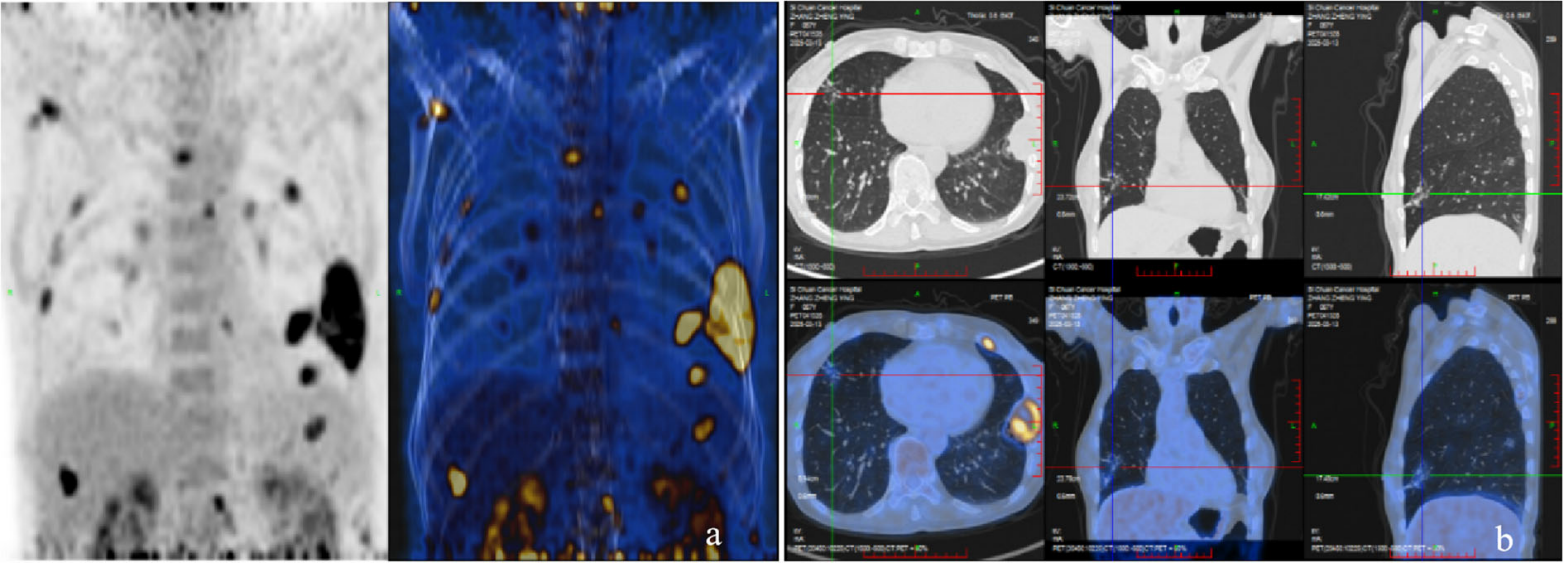
The overall condition **(a)** and condition of chest lesions **(b)** of the patient on PET CT.

**Figure 2:**
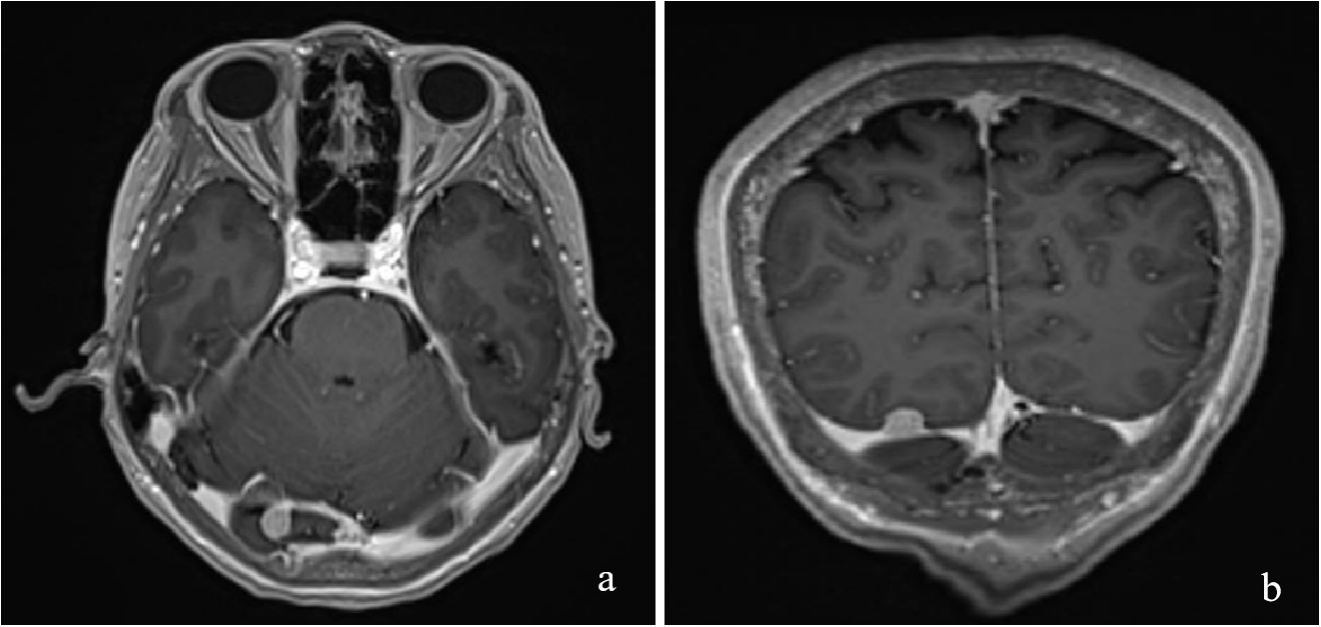
Lesions shown on the head MRI:coronal view **(a)** and axial view **(b)**.

**Figure 3:**
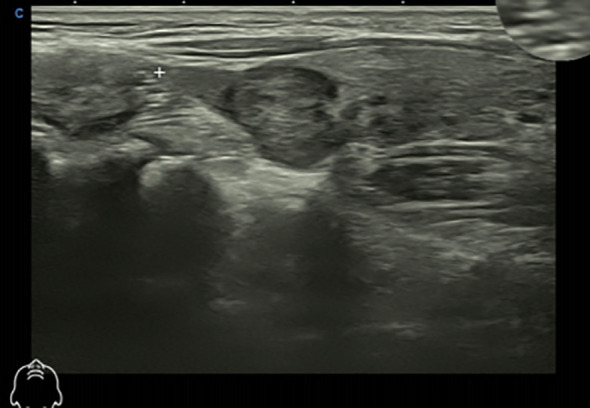
The patient’s color ultrasound showed hypoechoic nodules in the upper pole of the left lobe of the thyroid gland.

**Figure 4:**
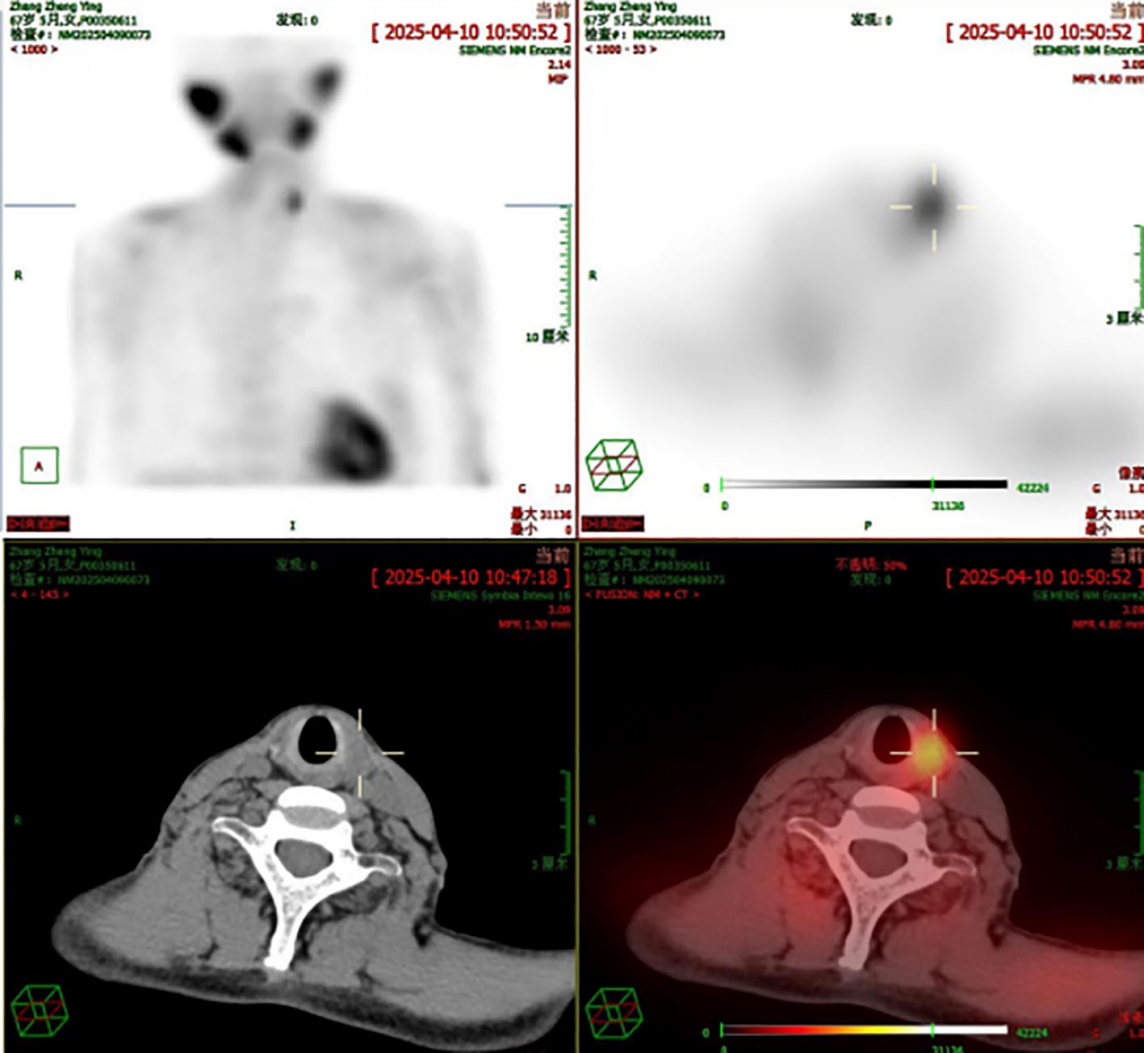
SPECT scintigraphy of the patient.

In March 2025, the patient underwent contrast-enhanced chest computed tomography (CT), which revealed multiple areas of bone destruction in the right scapula, sternum, and bilateral ribs, along with local soft tissue density shadows. The patient was referred to the Department of Medical Oncology for diagnostic confirmation, and a whole-body positron emission tomography CT (PET-CT) was performed. PET-CT results showed unevenly reduced bone mineral density, with recurrent osteolytic destruction involving multiple bilateral ribs, the right scapula, sternum, and L1 vertebral body. Notably, the axillary segment of the left sixth rib was identified as the focal point of destruction, with local soft tissue formation measuring approximately 46 × 36 mm. Increased uptake was observed in the local convexity of the left lung, with a maximum SUV of 9·5, and the density of the bilateral humeral bone marrow cavities was unevenly reduced (**Figure 1**). Cranial magnetic resonance imaging (MRI) revealed a right occipital lobe enhancing nodule with slightly higher T2-weighted imaging (T2WI) signal and a lower T1-weighted imaging signal, measuring approximately 1·1×0·8 cm, with poorly defined margins relative to adjacent meninges. Several T2WI hyperintense nodules were also observed in the occipital cistern, with the largest measuring approximately 0·9×0·6 cm (**Figure 2**). Based on these findings, metastases were initially suspected. The patient was admitted to the Department of Thoracic Surgery and underwent ultrasound-guided needle biopsy of a mass in the left chest wall. Ultrasound detected a cystic-solid mixed echogenic mass measuring approximately 7·8 x 3·0 cm, predominantly solid, with clear boundaries and irregular morphology. Pathology from the biopsy suggested giant cell-rich lesions consistent with brown tumors secondary to hyperparathyroidism, which are benign bone lesions caused by excessive osteoclastic bone resorption induced by sustained parathyroid hormone elevation. These findings prompted a multidisciplinary team discussion. Immunophenotype of tumor cells: CKpan (AE1/AE3) (-), H3.3G34R (-), H3.3G34V (-), H3.3G34W (-), P63 (partial +), SATB2 (partial +), Ki-67 (approximately 5%). Given the diagnostic uncertainty, the case was discussed at an MDT meeting. During multidisciplinary assessment, the imaging department noted that the patient’s brain MRI showed a right occipital lobe enhancing nodule; based on location and morphology, a meningioma was considered more likely. Concurrently, the Pathology team identified no histopathological evidence of malignancy, recommending differentiation between metabolic diseases and secondary lesions. The sonography team reported a nodule of unknown origin in the left lung lobe and advised repeating neck ultrasonography and performing parathyroid nuclide scintigraphy to assess its nature. Neck ultrasonography revealed a hypoechoic nodule in the upper pole of the left thyroid lobe, measuring approximately 19 mm ×8 mm ×16 mm, with well-defined borders, and irregular shape (**Figure 3**). Laboratory testing showed a PTH level of 1911·97 pg/mL, which was 21 times higher than the upper limit of normal. The serum calcium level was 3·07 mmol/L, the serum phosphorus level was 0·55 mmol/L, and the alkaline phosphatase level was 547 U/L, respectively. These results suggested significant parathyroid dysfunction. Therefore, we used nuclear medicine to further clarify the diagnosis of these patients. The results of single-photon emission CT/CT (SPECT-CT) parathyroid scintigraphy demonstrated an upper low-density nodule in the left thyroid lobe that was not clearly demarcated from the thyroid parenchyma. Uptake of 99mTc-methoxyisobutylisonitrile (99mTc-MIBI) was increased, suggesting hyperfunctioning parathyroid tissue (**Figure 4**).

### Diagnostic assessment

After multidisciplinary consultation, parathyroid carcinoma was initially considered given the markedly elevated PTH level and extensive skeletal involvement. However, the final diagnosis of multiple osteolytic bone lesions secondary to a parathyroid adenoma was established based on the absence of invasive histological features, a low Ki-67 index, and confirmatory immunohistochemical findings. Doctors in the Department of Head and Neck Surgery stated that surgical removal of the parathyroid tumors could significantly alleviate the patient’s symptoms and potentially achieve a curative outcome. The thoracic surgeon advised that thoracic surgery was not required at this stage, as bone destruction secondary to lung cancer was not suspected. Post-parathyroidectomy, the patient’s chest pain improved markedly. It was noted that if the rib bone destruction did not improve after surgery, thoracic surgery and related interventions should be reconsidered. The consensus decision was to proceed with left-sided parathyroidectomy, followed by re-evaluation to determine any further treatment needs.

### Therapeutic intervention

The patient underwent left upper parathyroidectomy and partial left thyroid lobectomy, which was performed because preoperative fine-needle aspiration of the adjacent thyroid nodule demonstrated benign follicular features and surgical exposure was required. Central neck dissection was not performed because no invasive features suggestive of parathyroid carcinoma were identified intraoperatively, and definitive postoperative pathology confirmed a benign parathyroid adenoma. Intraoperative frozen section analysis suggested a parathyroid adenoma; however, the definitive diagnosis was established based on permanent paraffin sections and immunohistochemical confirmation. The post-frozen-section paraffin pathological report demonstrated the following findings for the left superior parathyroid mass (the lesion): it presented as an irregular grayish-red tissue measuring 2 cm × 1.5 cm × 1.6 cm in dimensions, with a medium-textured cut surface. Immunohistochemical staining revealed positive expression of Syn, CgA, and PTH, negative expression of TTF-1, and a Ki-67 proliferation index of 1%. Based on these results, the lesion was diagnosed as a parathyroid adenoma. Notably, the intraoperative PTH level was recorded at 159·91 pg/mL, demonstrating a significant decline from preoperative levels. No specific preoperative medical treatment was administered to address the moderate hypercalcemia. Following surgical removal of the parathyroid lesion, serum calcium levels normalized promptly, as confirmed by postoperative laboratory testing. Postoperatively, the patient experienced a sore throat with cough and sputum production, which was aggravated during swallowing, but no symptoms such as numbness or carpopedal spasms were reported. Symptomatic treatments included mechanically assisted expectoration, red light therapy, calcium gluconate supplementation, and bromhexine for sputum reduction. On postoperative day two, follow-up measurements revealed a PTH level of 4·63 pg/mL, serum calcium level of 2·30 mmol/L, and serum phosphorus level of 0·52 mmol/L. By postoperative day four, the patient recovered well and was discharged from the hospital.

### Follow-up and outcomes

Two weeks after surgery, the patient’s rib pain had markedly improved. Laboratory review showed a serum calcium level of 1.98 mmol/L, serum phosphorus level of 0·83 mmol/L, and total 25-hydroxyvitamin D level of 17·52 ng/mL. At the last follow-up, the patient was in good general condition, with near-complete resolution of pain and other symptoms, and continued oral calcium supplementation.

## Discussion and conclusions

This case represents a relatively uncommon clinical scenario characterized by multiple skeletal lesions coexisting with PHPT. The patient presented with cough, sputum production, and left-sided rib pain, without clinical evidence of nephrolithiasis, gastrointestinal abnormalities, or neurological deficits. Given the CT findings of rib destruction with extension into the thoracic cavity, there was a considerable risk of misdiagnosis as primary pulmonary malignancy with osseous metastases.

Accurate differential diagnosis of this condition is critical, necessitating a multidisciplinary approach involving medical oncology, endocrinology, thyroid surgery, orthopedics, radiology, and pathology. The patient’s initial head MRI suggested possible metastasis; however, after multidisciplinary consultation, a meningioma was considered. This underscores the importance of multidisciplinary discussions. Distinguishing between benign parathyroid lesions and parathyroid carcinoma (PC) is also crucial for the diagnosis and treatment of primary hyperparathyroidism (PHPT). When the parathyroid hormone (PTH) level exceeds 1000 pg/mL, parathyroid carcinoma must be highly suspected. In this case, the patient’s preoperative PTH was approximately 1900 pg/mL, which raised such concerns at the time. Single-photon emission computed tomography-computed tomography (SPECT-CT) is helpful for the identification of parathyroid adenomas. The most commonly used parathyroid scintigraphy (SPECT) employs dual-phase acquisition of technetium-99m methoxyisobutyl isonitrile (^99m^Tc-MIBI), consisting of an early phase (at 20 minutes) and a delayed phase (at 120 minutes). Due to the different metabolic rates of ^99m^Tc-MIBI in normal versus hyperfunctioning parathyroid tissue, it is preferentially taken up and retained by the hyperfunctioning glands, whereas uptake by normal parathyroid tissue is extremely low, thereby facilitating lesion detection. The fusion of functional and anatomical imaging in nuclear medicine has a synergistic effect, and its diagnostic value far exceeds the sum of single-modality imaging. Furthermore, the patient’s PET-CT indicated no metastases, and intraoperative and postoperative PTH levels decreased significantly. Immunohistochemistry (Synaptophysin positive, Chromogranin A positive, Parathyroid Hormone positive, Ki-67 proliferation index 1%, and absence of invasive features) further ruled out parathyroid carcinoma, confirming the lesion to be benign.

Currently, the classic imaging features of advanced PHPT are rarely observed. Instead, the most consistent findings are biochemical abnormalities such as hypercalcemia and hypophosphatemia, with generalized osteopenia being the most common imaging manifestation. Gupta et al. (4) and Lu et al. (5) reported that parathyroid adenoma can be diagnosed using a combination of hematology, imaging, bone marrow aspiration, nuclear medicine, and other examinations. Marienhagen (6) and Gill (7) similarly documented cases of hypercalcemia crises stemming from PHPT, in which surgical intervention significantly reduced serum calcium levels. These cases highlight that the clinical symptoms of PHPT are often atypical, predisposing to misdiagnosis, and require prompt recognition and intervention. Studies have shown that PHPT affects both cortical bone and cancellous bone, increasing fracture risk (8). Currently, the two main treatment modalities are pharmacological and surgical. Pharmacologic management, generally reserved for patients who are not surgical candidates, includes bisphosphonates or denosumab to improve bone density. Vitamin D supplementation may be used in patients with deficiency, while estrogen therapy is less commonly employed today. However, surgery has been reported to achieve a biochemical cure and significantly increase bone mineral density in most patients compared with medical therapy, while being associated with a lower complication rate (9).

Once a parathyroid adenoma is diagnosed, surgical excision should be considered the first-line treatment. After tumor removal, intraoperative PTH levels are significantly reduced and return to normal within 24 hours postoperatively. Serum calcium concentrations gradually normalize, and the patients usually experience marked symptomatic relief and significant improvement in bone health.

### Patient perspective

I am a 67-year-old woman who was previously in relatively good health. I developed a persistent cough and severe chest pain that disrupted my daily life and sleep. Initial imaging tests at the hospital revealed extensive osteolytic lesions, and I was initially suspected of having malignant bone metastases possibly from lung cancer, which filled me with severe fear and anxiety. Through multidisciplinary evaluation including detailed laboratory tests and advanced imaging, I was finally diagnosed with primary hyperparathyroidism caused by a parathyroid adenoma, not cancer. This brought me immense relief, even though I was confused about this condition at first. After the doctors explained my condition clearly, I underwent surgery to remove the parathyroid adenoma. The surgery and recovery went smoothly, and my symptoms improved significantly within a few weeks: my chest pain eased, my cough subsided, and I gradually resumed the daily activities I had been unable to do before.

## Data Availability

The original contributions presented in the study are included in the article/supplementary material. Further inquiries can be directed to the corresponding author/s.
